# Evaluation of a Deep Learning Reconstruction for High-Quality T2-Weighted Breast Magnetic Resonance Imaging

**DOI:** 10.3390/tomography9050152

**Published:** 2023-10-18

**Authors:** Timothy J. Allen, Leah C. Henze Bancroft, Orhan Unal, Lloyd D. Estkowski, Ty A. Cashen, Frank Korosec, Roberta M. Strigel, Frederick Kelcz, Amy M. Fowler, Alison Gegios, Janice Thai, R. Marc Lebel, James H. Holmes

**Affiliations:** 1 Department of Medical Physics, University of Wisconsin-Madison, 1111 Highland Avenue, Madison, WI 53705, USA; 2 Department of Radiology, University of Wisconsin-Madison, 600 Highland Avenue, Madison, WI 53792, USA; 3 GE Healthcare, 3000 N Grandview Blvd, Waukesha, WI 53188, USAmarc.lebel@ge.com (R.M.L.); 4 Carbone Cancer Center, University of Wisconsin-Madison, 600 Highland Avenue, Madison, WI 53792, USA; 5 Department of Radiology, University of Iowa, 169 Newton Road, Iowa City, IA 52242, USA; 6 Department of Biomedical Engineering, University of Iowa, 3100 Seamans Center, Iowa City, IA 52242, USA; 7 Holden Comprehensive Cancer Center, University of Iowa, 200 Hawkins Drive, Iowa City, IA 52242, USA

**Keywords:** deep learning, image reconstruction, breast MRI, high resolution, reader study, SNR

## Abstract

Deep learning (DL) reconstruction techniques to improve MR image quality are becoming commercially available with the hope that they will be applicable to multiple imaging application sites and acquisition protocols. However, before clinical implementation, these methods must be validated for specific use cases. In this work, the quality of standard-of-care (SOC) T2w and a high-spatial-resolution (HR) imaging of the breast were assessed both with and without prototype DL reconstruction. Studies were performed using data collected from phantoms, 20 retrospectively collected SOC patient exams, and 56 prospectively acquired SOC and HR patient exams. Image quality was quantitatively assessed via signal-to-noise ratio (SNR), contrast-to-noise ratio (CNR), and edge sharpness. Qualitatively, all in vivo images were scored by either two or four radiologist readers using 5-point Likert scales in the following categories: artifacts, perceived sharpness, perceived SNR, and overall quality. Differences in reader scores were tested for significance. Reader preference and perception of signal intensity changes were also assessed. Application of the DL resulted in higher average SNR (1.2–2.8 times), CNR (1.0–1.8 times), and image sharpness (1.2–1.7 times). Qualitatively, the SOC acquisition with DL resulted in significantly improved image quality scores in all categories compared to non-DL images. HR acquisition with DL significantly increased SNR, sharpness, and overall quality compared to both the non-DL SOC and the non-DL HR images. The acquisition time for the HR data only required a 20% increase compared to the SOC acquisition and readers typically preferred DL images over non-DL counterparts. Overall, the DL reconstruction demonstrated improved T2w image quality in clinical breast MRI.

## 1. Introduction

The American Cancer Society estimates that one out of eight women will develop breast cancer in their lifetime [1]. MRI plays an important role in breast cancer care. MRI is recommended for breast cancer screening in high-risk populations, evaluating extent of disease, and as the most accurate method to assess response to neoadjuvant therapy [2,3,4]. Typical MRI protocols for breast imaging include the following acquisitions: T1-weighted (T1w) dynamic contrast enhanced (DCE), T2-weighted (T2w), and T1w without fat saturation. Diffusion-weighted imaging may also be included. Of these acquisitions, DCE MRI provides the greatest sensitivity for detecting breast cancer [5,6,7]. T2w imaging is used in tandem with DCE MRI to detect T2 hyperintense findings, such as cysts, lymph nodes, or edema. T2 hyperintensity helps radiologists to differentiate benign from malignant findings; benign findings, such as fibroadenomas, are more likely to appear hyperintense on T2w images whereas malignant findings are most often not T2 hyperintense.

T2w imaging requires long repetition times, leading to time-consuming acquisitions, which can result in patient discomfort and motion-related artifacts. Breast radiologists generally prefer to compromise on signal-to-noise ratio (SNR) and spatial resolution in favor of shorter acquisition times to reduce patient motion. While T1w DCE images frequently achieve sub-millimeter in-plane spatial resolution, T2w imaging is often limited to resolutions well above one millimeter. This makes T2w images comparatively blurry with a decrease in spatial detail with volume averaging of small findings. Attempting to increase the spatial resolution while maintaining a reasonable acquisition time results in prohibitively low SNR.

Machine learning (ML) and the sub-field of deep learning (DL) may help minimize image quality compromises in T2w breast MRI. ML and DL have led to many innovations and opportunities in the field of medical imaging [8,9]. DL-based reconstruction algorithms are particularly impactful and help to improve medical images via noise reduction, resolution enhancement, and/or artifact suppression [10]. DL reconstructions have been investigated for neurological [11], musculoskeletal [12], abdominal [13], and cardiac MRI imaging [14]. Despite their promising results throughout the body, application of DL reconstructions to breast imaging has been lacking. ML and DL research in breast MRI has largely focused on DL to analyze existing images through breast segmentation, lesion segmentation, lesion classification, and prognosis as discussed in multiple review articles on the topic [15,16]. Only recently have publications emerged focused on DL reconstructions in breast MRI and have been mostly confined to diffusion-weighted imaging [17,18]. One paper by Yang et al. sought to improve T2w imaging of the breast using a 3D acquisition and compressed sensing reconstruction with a DL-based sparsifying transform [19].

The paucity of research into DL reconstructions for breast MRI may be due, in part, to the need for a large, diverse set of high-quality MR exams to serve as the ground truth. Compiling such a dataset is especially challenging in breast MRI due to the extensive anatomical variability including differences in breast size, density of fibroglandular tissue, presence of implants, implant type, and tissue distribution. Many patients also have post-surgical changes (e.g., mastectomy) adding further complexity. Even with sufficient anatomical variability, the training data would ideally encompass the range of expected acquisition parameters (TE, TR, parallel imaging, matrix size, etc.) and hardware configurations (magnet strength, receive coil, scanner model, etc.). Without this diversity, a change in acquisition protocol might require a complete retraining of the DL algorithm which is unfeasible in a typical clinical setting. Even with an existing, fully trained algorithm, implementing these methods for clinical usage would pose many technical challenges requiring specialized personnel, data transfer systems, and dedicated computing resources.

A general-purpose DL reconstruction recently introduced by Lebel offers a relatively accessible approach that has been shown to offer reduced noise, decreased truncation artifact, and enhanced edge sharpness [20]. Rather than being developed on and trained for a specific anatomy or acquisition protocol, it was trained on a dataset of 4 million image pairs encompassing a broad variety of image content and contrasts. In theory, this makes the method more flexible with less dependence on the specific anatomy and acquisition, thus reducing the need for curation of extensive training data and the time-consuming re-training process for each specific use case. It is, however, limited to 2D MR sequences. Already, this DL reconstruction has shown improved image quality and better clinical task performance in a sub-set of anatomic settings, including pituitary [21], cardiac [22], prostate [23], and orthopedic hip and shoulder imaging [24]. Notably, the model was not found to require re-training to achieve increased image quality in these scenarios. Recently, the DL reconstruction was made available as a commercial product under the tradename AIR Recon DL (GE Healthcare), allowing for ease of implementation in a clinical setting.

While the existing research using Lebel’s algorithm in other anatomies and contrast is promising, breast T2w imaging is unique. Namely it provides unique morphology and contrast that are distinct from the previously tested imaging settings since it potentially has very sparse areas of fibroglandular tissue over a dark, fat-suppressed background. Thus, performance of the DL algorithm in the setting of T2w breast MR imaging is currently unknown and requires further validation. We hypothesize that Lebel’s DL reconstruction method will improve T2w imaging of the breast for standard-of-care protocols and that it will allow increased spatial resolution while limiting SNR losses for breast radiologists.

The purpose of this work is to evaluate the performance of an existing general-purpose DL reconstruction prototype in the setting of T2w breast imaging. The performance is tested with multiple acquisitions consisting of an existing standard-of-care (SOC) T2w breast protocol and a tailor-made high-spatial-resolution (HR) protocol.

## 2. Materials and Methods

### 2.1. MRI Protocols

A summary of T2w protocols used in this study is provided in Table 1. Imaging was performed on one of two 3.0T MR scanners: (1) SIGNA Premier (GE Healthcare, Waukesha, WI, USA) or (2) Discovery MR750w (GE Healthcare). Breast-specific 16-channel receive array coils were used (Sentinelle, Dunlee, The Netherlands). The SOC T2w acquisition was performed with a 2D fast spin echo (FSE) sequence with an echo train length of 16. Imaging parameters included a 288 × 288 acquired in-plane matrix with 2 mm slices and a variable field of view (32 cm to 36 cm) as required to achieve full coverage of the breasts and axilla while maximizing spatial resolution in smaller patients. Parallel imaging was performed with an acceleration factor of 3 (ASSET on scanner 1 or ARC on scanner 2, GE Healthcare). The difference in parallel imaging technique between the two MRI scanners was necessary to avoid changes to our institution’s clinical imaging protocols on these two MRI systems. An HR acquisition was created from the SOC T2w sequence by increasing the acquisition matrix size to 448 × 448 and increasing the parallel imaging acceleration factor from 3 to 4. The 448 × 448 matrix size results in an in-plane resolution that matches the T1w DCE acquisition performed routinely at our institution. The parallel imaging increase was implemented to reduce the time penalty resulting from the increased number of phase-encoding lines. The mean acquisition times for the SOC acquisition were 247 or 308 s and the HR acquisition had mean times of 237 and 284 s for scanners 1 and 2, respectively.

### 2.2. Deep Learning Reconstruction

The DL reconstruction used in this study was a vendor-supplied prototype (GE Healthcare) and has been previously described by Lebel [20]. While this work uses a prototype version, a commercial version has since been released (AIR Recon DL, GE Healthcare). This reconstruction is based on a convolutional neural network and is trained to reduce image noise, increase image sharpness, and reduce the prevalence of simple artifacts such as truncation ringing. The convolutional network was pre-trained by the vendor using supervised learning consisting of pairs of pristine and typical datasets. No additional training was performed as part of this study. The DL algorithm does not replace the conventional reconstruction pipeline; rather, it works as a single step within that pipeline. Specifically, it disables standard k-space apodization filters and zero-padding interpolation used in conventional reconstructions in favor of a feed-forward network that interpolates images and suppresses random noise while remaining consistent with the acquired data. The level of denoising performed by the reconstruction is determined by a user-controlled parameter which relates to the amount of estimated noise variance to be removed from the acquired images. For the prototype version used in this work, the available settings for this parameter are 0% (no noise removal), 25%, 50%, 75%, and 100% (complete removal of estimated noise). Throughout this manuscript, the following shorthand will be used for these different denoising settings: DL0, DL25, DL50, DL75, and DL100, respectively. The commercial version allows noise reduction settings of 25%, 50%, and 75% only. It has been suggested to avoid using DL100 to allow for a controlled level of remaining noise to preserve the expected appearance of the images [24]. It should be noted that DL0 is distinct from the conventional reconstruction pipeline as it still attempts to sharpen images and prevent truncation artifacts.

To generate DL-enhanced images for this study, raw k-space data were collected and saved. The raw data were transferred to an MR scanner with the DL reconstruction prototype installed. There, the denoising setting could be set and the reconstruction executed using the scanner reconstruction hardware/software. This ensured that the only difference between our images and clinically obtained images was the DL reconstruction component. Non-DL images were generated using the conventional on-scanner product reconstruction pipeline.

### 2.3. Technical Analysis in Phantoms

Phantom experiments were conducted to assess the hypothesized denoising and sharpness improvements using the DL algorithm. An ACR accreditation phantom (J.M. Specialty Parts, San Diego, CA, USA) was imaged on scanner 1 using an 11-channel head and neck coil (GE Healthcare). This coil was used rather than a breast coil to accommodate the geometry of the phantom. Imaging was first performed using the SOC T2w acquisition. Then, parallel imaging was increased from 3 to 4 while the acquisition matrix was systematically increased from 288 × 288 to 448 × 448, corresponding with acquisition pixel sizes of 1.0 mm^2^, 0.9 mm^2^, and 0.8 mm^2^ at a 32 cm field of view (320 × 320, 356 × 356, and 400 × 400). Images were reconstructed from the raw data using varying denoising settings of the DL reconstruction ranging from DL0 to DL100.

SNR and image sharpness of the resulting images were measured on multiple slices near the center of the phantom. SNR was expected to increase when a noise reduction setting of greater than 0% was used. Measurements were performed using the subtraction method described by NEMA [25]. Sharpness was measured using the edge response at two vertical and two horizontal edges within a single central slice of the phantom. Sharpness was defined as the width of the edge measured from 20% to 80% of the signal intensity. The final size of DL reconstructed images depends on the acquisition matrix (see discussion). Thus, a discrepancy between the DL images size and the non-DL image size could exist. In such cases, a second non-DL reconstruction with an increased reconstruction matrix size was obtained through an offline version of the conventional vendor reconstruction.

### 2.4. Retrospecive SOC Imaging

All human subject data were collected under IRB approval and are HIPAA compliant. T2w series from twenty consecutive clinical breast MR exams on a Discovery MR750w (scanner 2) were retrospectively collected. Any breast MRI (with or without intravenous contrast) including a T2w acquisition was eligible. The T2w images were always collected prior to contrast administration. Using the raw acquisition data, non-DL and DL T2w images series with noise reduction settings ranging from DL0 to DL100 were reconstructed as described in Section 2.2.

#### 2.4.1. Quantitative Assessment

Contrast-to-noise ratio (CNR) measurements were performed in all SOC images. Due to the challenges of finding consistent anatomic fibroglandular tissue features between subjects, the CNR between muscle and suppressed breast fat was used as a surrogate. For each subject, two regions of interest were placed using the non-DL image series: (1) within the pectoralis major muscle; (2) within an area of uniformly fat suppressed fat. These regions were then copied to each corresponding DL image. The CNR was calculated as the difference in mean signal between muscle and fat regions divided by the standard deviation in the fat region. Contrast (independent of noise) was also measured.

#### 2.4.2. Qualitative Assessment

This work included a set of qualitative metrics to directly assess radiologist’s perception of image quality. This qualitative assessment was performed using a reader study consisting of comparing the image quality of non-DL images and DL75 images. A noise reduction level of DL75 was chosen as it has been demonstrated to produce high image quality in previous studies [21,22,23,24]. Four radiologists participated in the reads: one board-certified, fellowship-trained breast radiologist with 15 years of experience (R.S.); a board-certified breast radiologist with 25 years of experience (F.K.); two radiologists who were undergoing breast fellowship training at the time of the study (A.G. and J.T.). Each radiologist independently viewed the non-DL and DL-enhanced SOC series in a side-by-side manner. Readers were blinded to image reconstruction type and allowed to scroll through the 3D image volume. Image order was not randomized. Image scoring was based on four image quality criteria: (1) presence of artifacts, (2) perceived SNR, (3) perceived sharpness, and (4) overall image quality. The word “perceived” is used here to distinguish the radiologists’ perception of SNR and sharpness from the technical measurements that were performed in phantom experiments. Each image quality category was scored using a 5-point Likert scale with 1 indicating poor performance and 5 indicating excellent performance. The rubric for overall image quality is shown in Table 2 with the remaining categories included in the Supplementary Materials (Table S1). Notably, the scoring criteria included language asking readers to assess whether poor category performance impacted the diagnostic utility of the image. If the readers felt the diagnostic capability was impacted, then they were instructed to score the performance no higher than 2 for that category. Wilcoxon signed-rank non-parametric tests were used to test for differences in average image quality scores between non-DL and DL75 images using a significance level of *p* = 0.05.

Readers were also asked to state an overall preference for either non-DL or DL75 images on a case-by-case basis. Responding “no preference” was allowed. Further, since signal intensity in T2w breast is used to assist interpretation of DCE findings, one task-based question was designed to mimic the clinical interpretation of T2w breast images. Readers were asked to identify hyperintense features in the T2w series (e.g., cysts, lymph nodes, edema, etc.) and compare the signal intensity between the non-DL and DL75 images. The relative signal intensity between the non-DL and DL75 images, as perceived by the radiologists, was recorded as either brighter in non-DL, brighter in DL75, or equally as bright.

### 2.5. Prospective SOC and HR Imaging

Fifty-six patient volunteers were prospectively imaged using a research T2w sequence added to their clinical exam alongside the SOC T2w series. Prospective data were collected only after subjects provided informed consent. Patients were imaged on either scanner 1 or scanner 2. Individuals with implants were excluded because, at our institution, these patients already have additional implant-specific sequences performed as part of their SOC, lengthening the exam. The HR data were acquired immediately after the SOC T2w acquisition and prior to contrast agent injection. Raw SOC and HR data were used to reconstruct non-DL and DL T2w image series across the range of denoising levels (DL0–DL100).

Similar to the approach for the retrospective T2w image data, the prospective image data were analyzed using a combined quantitative and qualitative approach. For the quantitative assessment, muscle–fat CNR measurements were obtained for the SOC and HR images using non-DL reconstruction and DL reconstructions across the range of denoising levels. For the qualitative assessment, a second reader study was performed. Three image series were read: (1) the non-DL SOC T2w series, (2) the non-DL HR T2w series, and (3) the HR T2w with DL75. Two board-certified, fellowship-trained breast radiologists (R.S. and A.F.) with 15 and 13 years of experience, respectively, scored all cases independently. Readers were asked to identify a preferred series in each case. Stating no preference was allowed. Again, Wilcoxon signed-rank non-parametric tests were used to test for significance difference between the three series.

## 3. Results

### 3.1. Phantom Experiments

**Figure 1:**
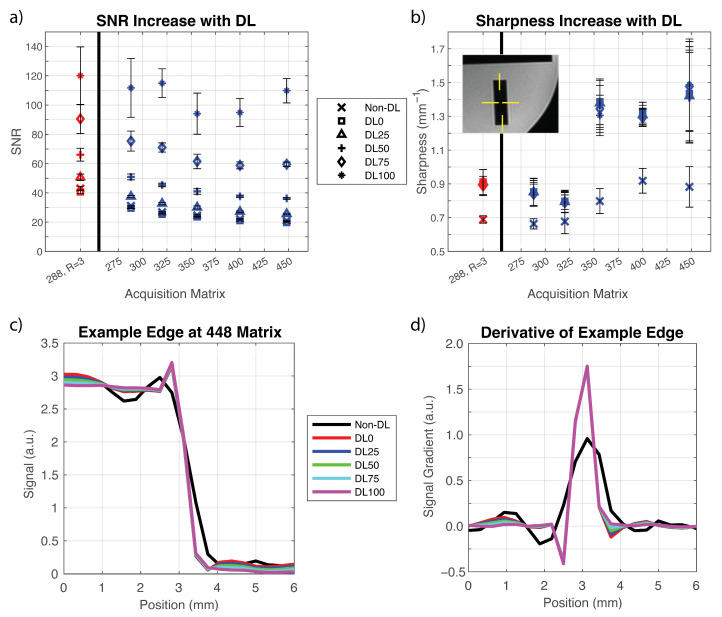
Application of a deep learning (DL) reconstruction provided an increase in SNR and image sharpness as measured in a phantom. (**a**) SNR measurements show a non-linear increase with DL denoising level. The measured increase in SNR is consistent across the difference acquisition matrix sizes and acceleration factors. Red points were acquired using an acceleration factor of R = 3 which matches the standard-of-care (SOC) T2w breast protocol used in this work. Blue points use an acceleration factor of R = 4. SNR is averaged across multiple slices. Error bars show one standard deviation. (**b**) Image sharpness increased 1.2 to 1.7 times when DL was applied. This increase was independent of denoising level. Points are average sharpness measurements obtained from four edges of phantom imaged with the high-resolution protocol (inset). The increase in sharpness is more pronounced at higher acquisition matrices. Error bars represent standard deviation of measured sharpness. (**c**) Example edge from phantom demonstrating edge sharpening and a reduction in truncation artifact. (**d**) Derivative of the edge from (**c**).

Application of the DL reconstruction to the phantom data resulted in a 1.2 times increase in the SNR for a denoising level of DL25, a 1.5 to 1.7 times increase for DL50, a 2.1 to 2.8 times increase for DL75, and a 2.8 to 5.2 times increase for DL100 (Figure 1a). Within these ranges, SNR increased with increasing acquisition matrix size. Using a denoising level of DL0 decreased the SNR to 0.95 times its value for the non-DL images. Edge sharpness was increased by 1.2 to 1.3 times for acquisition matrices from 288 × 288 to 320 × 320 and 1.4 to 1.7 times for matrices of 356 × 356 and above (Figure 1b). The effect of image sharpening was consistent across all denoising levels tested (DL0–DL100). An example of the edge response is shown in Figure 1c,d, which visually shows a reduction in truncation artifact as well as the increased sharpness with the DL reconstructions as compared to the conventional non-DL reconstruction.

### 3.2. Retrospective SOC Imaging

**Figure 2:**
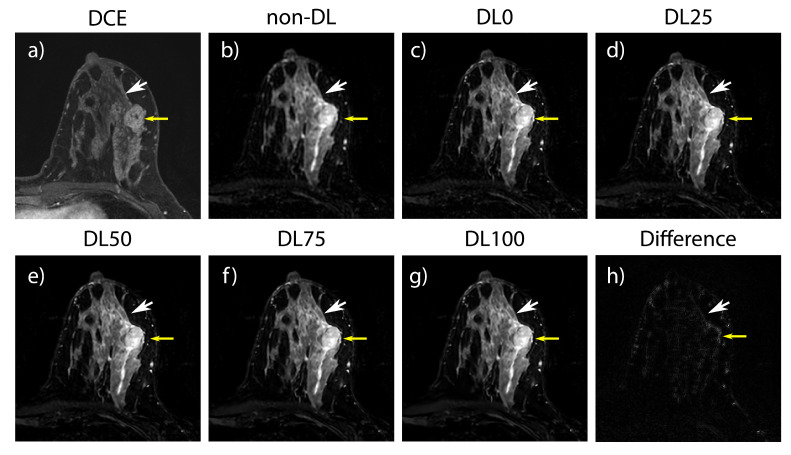
A 37-year-old female who underwent breast MRI for high-risk screening. Left breast demonstrates a biopsy-proven benign fibroadenoma with intermediate signal on the T2w images (yellow arrow, small arrowhead). (**a**) Dynamic-contrast-enhanced T1w image (DCE) showing fibroadenoma enhancement (yellow arrow, small arrowhead). (**b**) Standard-of-care (SOC) T2w acquisition without deep learning reconstruction (DL). (**c**–**g**) Same raw data as (**b**) but with DL reconstruction with various noise reduction levels (DL0-DL100). Images generated with the DL reconstruction show reduced image noise and enhanced edge sharpening helping to visualize fine fibroglandular tissue details (white arrow, large arrowhead). (**h**) Difference image showing the absolute value of the difference between the images shown in (**b**,**f**), demonstrating reduction in noise and increased edge sharpness.

**Figure 3:**
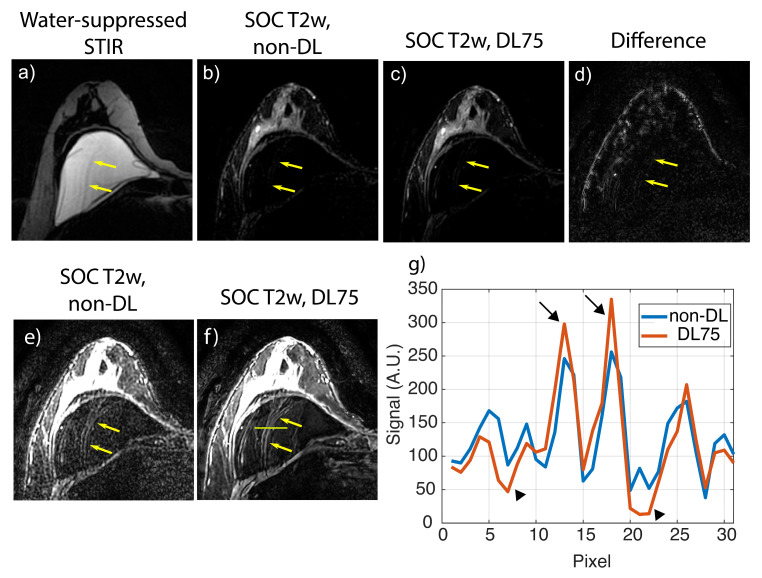
Images from a 65-year-old female with bilateral silicone implants. The deep learning reconstruction (DL) provides an apparent decrease in image noise and sharpening. In this example, a motion artifact is intensified within the implant (yellow arrows). (**a**) Water-suppressed STIR image showing silicone implant. (**b**) Standard-of-care (SOC) T2w acquisition without deep learning. (**c**) Same raw data as (**b**) but with DL75. (**d**) Difference image showing the absolute value of the difference between images (**b**,**c**). (**e**,**f**) Different image window and level display settings of the images from (**b**,**c**) highlighting the motion artifact (yellow arrows). (**g**) Profile across the motion artifact within the implant depicted as the horizontal line in (**f**). The black arrows show the increased signal and sharpness in the DL images while the black arrowheads show a reduction in signal surrounding the artifact.

**Figure 4:**
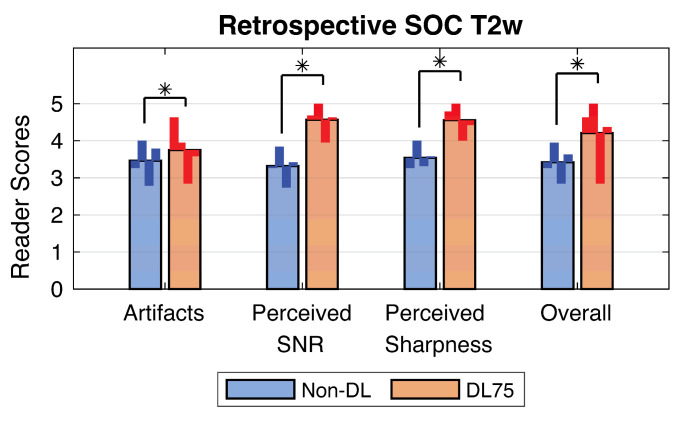
Comparison of mean image quality from 19 subjects read by 4 readers showing the deep learning (DL75) reconstructions scored significantly higher than non-DL images in each measured category. Stars indicate statistically significant differences with *p* < 0.05. The smaller, darker bars demonstrate individual averages for each reader.

Included in the collected SOC cases were two subjects with bilateral silicone implants, two with unilateral silicone implants, and one subject imaged during lactation. One subject was excluded because the data were unavailable due to an error in data transfer, leaving nineteen in the analysis. Example cases from the retrospective SOC T2w breast data are shown in Figure 2 and Figure 3. For the quantitative analysis, non-DL images had a muscle–fat CNR of 4.5 +/− 1.3 (mean +/− one standard deviation); DL0 was 4.3 +/− 1.3, DL25 was 5.1 +/− 1.6, DL50 was 6.2 +/− 2.1, DL75 was 8.1 +/− 3.3, and DL100 was 12.6 +/− 9.5. Boxplots showing the CNR distribution for each denoising level and non-DL images can be found in the Supplementary Materials (Figure S1a). Muscle–fat contrast remained the same regardless of reconstruction technique used (Figure S1b). Analysis of the reader study found that images reconstructed using DL75 had a statistically significant increase in each of the four tested categories (presence of artifacts, perceived sharpness, perceived SNR, and overall quality) when compared to the conventional non-DL reference images (Figure 4).

Of the 76 reader/subject combinations (19 patient MR cases with 4 readers each), the DL75 images were preferred in 71 instances (93%). Radiologists stated that the preference was due to the decreased image noise and increased sharpness. The non-DL images were preferred in four instances and no preference was found in one instance. The reasons given for preference of the non-DL images mentioned increased artifact intensity in DL75 images. Details of the preferences for each reader can be found in Table S2. T2-bright features in DL75 images had equal to or greater intensity than the same features in non-DL images as perceived by radiologist readers for 65 out of 76 cases (86%).

### 3.3. Prospective SOC and HR Imaging

**Figure 5:**
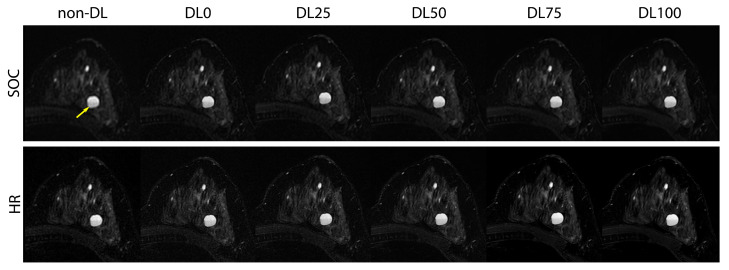
Images from a 48-year-old female undergoing high-risk screening MRI with multiple benign T2 hyperintense cysts. Complicated cyst with fluid–fluid level (yellow arrow) demonstrates the image sharpening from high spatial resolution (HR) imaging in combination with the deep learning reconstruction (DL). Note improved visualization of the cyst and surrounding fibroglandular tissue with increasing DL denoising level. SOC—standard-of-care (SOC) T2w. DL0-DL100: Deep learning with various levels of denoising.

**Figure 6:**
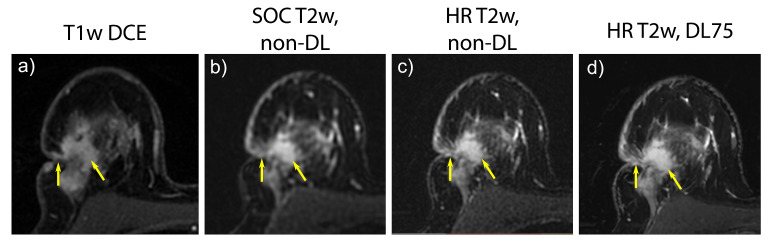
Images from a 52-year-old female with recurrent cancer and suspected skin invasion on T1w DCE and the corresponding T2w images showing edema (arrows). Skin invasion was later confirmed by pathology. High-spatial-resolution (HR) imaging allows for a more detailed visualization of the recurrent cancer and the associated skin retraction and edema at the cost of increased noise. Application of a deep learning reconstruction (DL) allows for a reduction in that noise allowing for high-resolution imaging with increased SNR. (**a**) Contrast-enhanced T1w image demonstrating lesion enhancement. (**b**) Standard-of-care (SOC) T2w, non-DL image. (**c**) HR T2w, non-DL image. (**d**) HR T2w, DL75 image.

HR protocol data from two subjects were excluded due to a technical and operator error during the pre-scan calibration procedure leaving 54 subjects in the analysis. The SOC sequences required an average acquisition length of 241 s while the HR required 293 s (20% increase) due to an increase in the number of phase encodings. Without the increase in parallel imaging acceleration factor from 3 to 4, the HR acquisition would have necessitated an estimated 374 s acquisition (55% increase). Example images from the HR acquisition can be found in Figure 5 and Figure 6.

**Figure 7:**
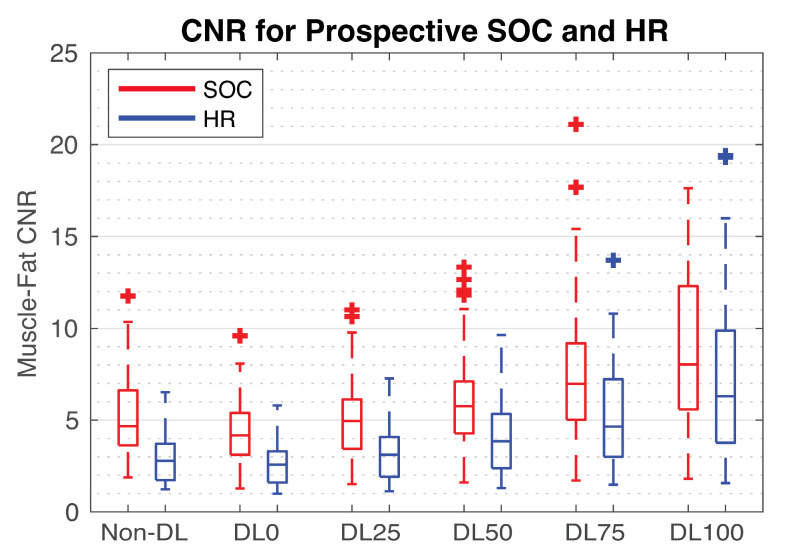
The deep learning (DL) reconstruction increased the muscle–fat contrast-to-noise ratio (CNR) when the denoising level was above 25% (DL25) for both standard-of-care (SOC) and high-resolution (HR) acquisitions. Use of DL without denoising (DL0) slightly reduced the CNR. The median CNR for the HR acquisition with DL75 was roughly equal to the SOC non-DL CNR. Demonstrated here using box plots are the distributions of CNR measurements for 54 prospectively acquired SOC and HR exams. The horizontal lines in the middle of the box plots represent the median value. Three outlier points existed for SOC DL100 between 30 and 40. These were cropped to preserve the readability of the plot.

Non-DL SOC images had a muscle–fat CNR of 5.3 +/− 2.2 (mean +/− one standard deviation); DL0 was 4.4 +/− 1.8, DL25 was 5.1 +/− 2.2, DL50 was 6.1 +/− 2.7, DL75 was 7.7 +/− 3.9, and DL100 was 10.0 +/− 7.0. Meanwhile, the mean HR CNR measurements were 3.0 +/− 1.3 for non-DL, 2.7 +/− 1.2 for DL0, 3.2 +/− 1.4 for DL25, 4.1 +/− 1.9 for DL50, 5.3 +/− 2.7 for DL75, and 7.2 +/− 4.2 for DL100. Boxplots showing the CNR distribution for each acquisition and reconstruction combination can be found in Figure 7.

**Figure 8:**
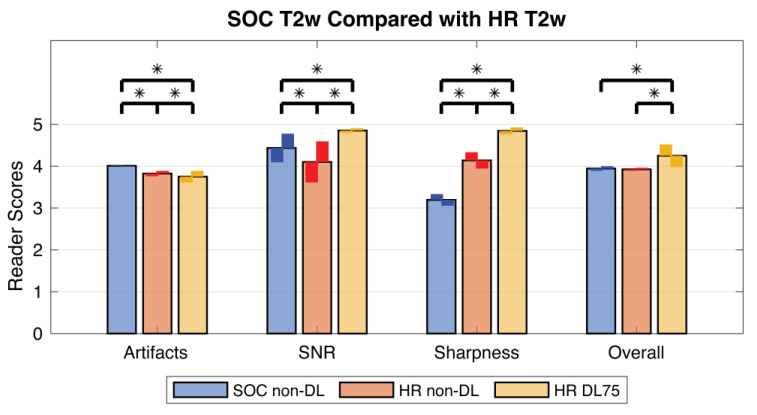
The high spatial resolution (HR) acquisition led to a significant increase in perceived image sharpness but at the cost of significantly worse scoring for both artifacts and perceived SNR. Subsequent application of the deep learning reconstruction (DL75) led to significantly higher perceived SNR with scoring exceeding that of the standard-of-care (SOC) images. Further, there was an additional increase in perceived sharpness and overall quality for HR images with DL reconstruction. Data from 54 subjects were evaluated by 2 radiologists. Stars indicate statistically significant differences in the scores with *p* < 0.05. The smaller, darker bars demonstrate individual averages for each reader.

Results of the image quality scoring are summarized in Figure 8. The non-DL HR images scored significantly higher in perceived image sharpness, but significantly worse in image artifacts and perceived SNR when compared to SOC images. No significant change was detected in overall quality (*p* = 1.0). Application of DL75 to HR data led to a significant increase in perceived sharpness, perceived SNR, and overall quality compared to non-DL HR images. However, DL75 HR images scored significantly worse in the artifact category. Comparing DL HR75 images to SOC images demonstrated a significant increase in SNR, sharpness, and overall quality but significantly worse performance in the artifact category.

Image preference was split between the two radiologists. One reader preferred the HR T2w images with DL75 in 50 of 54 cases. The reasons given were the decreased image noise and enhanced sharpness. The second reader was split between SOC T2w images (23 out of 54) and the HR images without DL75 (31 out of 54). Comments from this radiologist indicated that they felt the HR images with DL75 “looked too sharp” or had a “fake look to anatomy outside [the] breast”. Full results for the reader preference scoring can be found in Table S2.

## 4. Discussion

This work evaluated the performance of an extant, vendor-provided DL reconstruction for improving breast T2w image quality using both an SOC and an HR protocol. Overall, the DL-based reconstruction performed well with both protocols. Quantitative analysis of the phantom experiments validated the increase in perceived SNR and image sharpness from the DL reconstruction as assessed by the radiologists when using breast-specific acquisition parameters for increased acceleration factors and for increased matrix sizes. In vivo CNR measurements improved for both SOC and HR imaging when the DL reconstruction was used. Importantly, the improvements to the quantitative image quality metrics were also reflected in the qualitative scoring assessment from the radiologists. The HR DL75 images were scored higher than SOC non-DL images in terms of perceived sharpness, perceived SNR, and overall quality, and demonstrated higher CNR. The DL reconstruction appears to enable HR imaging without the typical tradeoff of SNR and a more modest increase in scan time (20% vs. 55%). The DL algorithm could potentially be used in a similar way with other T2w breast protocols that struggle with the balance between scan time, spatial resolution, and SNR. The increase in image quality may assist radiologists in their interpretation and allow better comparison between DCE images and T2w imaging; this, ultimately, is hypothesized to improve diagnostic capability and improve the specificity of MRI. Generally, this work demonstrated that the non-specific DL algorithm introduced by Lebel can be used to improve image quality in T2w breast MRI using a variety of acquisition protocols.

As is widely noted in the medical imaging field and recently affirmed in multiple ISMRM imaging challenges [26,27], assessments of image quality using qualitative vs. quantitative metrics can disagree. For this reason, this work chose to include both types of assessment and mirrors other existing works which investigates this DL reconstruction. Image sharpness measurements by Van der Velde et al. in cardiac MRI found that image sharpness was improved with the DL reconstruction but was consistent across the different denoising settings in agreement with our own findings [22]. Wang et al. also explored T2w imaging but focused on the clinical prostate imaging [23]. In prostate, the tissue fills most of the imaging volume and T2-bright features exist throughout most of the image. T2w breast imaging, in contrast, has a relatively sparse tissue distribution and few T2-bright features. Thus, breast provides a unique challenge for reconstruction. Both Wang et al. and Koch et al. found that the reconstruction improved image quality and reported that radiologists unanimously preferred DL images over non-DL images [23,24]. While this was true for the SOC acquisition in our study, and radiologists agreed that the DL reconstruction improved image quality with the HR acquisition, they disagreed on HR image preference. Koch et al. also noted a change in effective image contrast-to-noise ratio with denoising levels, which was similar in our study for breast tissue [24]. Our study has some similarities with that of Yang et al. [19], who also sought to improve T2w resolution for breast imaging. However, they used under-sampled 3D acquisition and compressed sensing reconstruction that included a DL-based sparsifying step to synthesize the missing k-space lines. We used 2D acquisition and Lebel DL reconstruction [20] to denoise and sharpen the images. Both techniques showed improved image quality according to both qualitative and quantitative assessment using HR acquisition combined with DL. However, it is difficult to directly compare the two studies due to the numerous differences in the choice for the baseline reference T2w imaging (different clinical standards), subject inclusion criteria, SNR/CNR measurement technique, and chosen qualitative assessment metrics.

In our work, relative signal intensity, as perceived by radiologists, remained consistent after the application of DL with T2-bright features retaining their hyperintensity in 86% of cases. Measured contrast was also consistent and independent of reconstruction type. Thus, it appears the contrast and, theoretically, the clinical utility of the images were preserved. Additionally, the breast radiologists in this study largely preferred the DL75 images over their non-DL counterparts when viewing data acquired with the SOC acquisition. While readers had a more mixed preference for the HR images, their preference was not tied to their image quality scoring. Together with the higher perceived quality, these findings indicate that the DL could potentially find acceptance as a clinically feasible reconstruction. However, this is contingent on future work demonstrating equivalent or superior diagnostic performance compared to the existing non-DL images. The DL reconstruction may provide sharper T2w images with increased perceived SNR that better assist interpretation of T1w DCE images. Since T2w images are typically used to help with discriminating between benign and malignant findings, this may help to improve the specificity of breast MRI.

In the phantom experiments, the SNR increased in a non-linear fashion as the denoising factor was increased, consistent with results by Lebel [20]. This work further showed that SNR increases were consistent across all the measured acquisition matrix sizes and acceleration factors. The SNR increase was slightly greater at larger acquisition matrix sizes, which is likely explained by the increased noise at smaller voxel sizes providing more opportunity for noise removal. When DL0 was used, the SNR was lower compared to the non-DL images. In the conventional reconstruction pipeline, some noise reduction is achieved by applying an apodization filter to the raw k-space data, thereby removing high frequency noise. In contrast, the DL pipeline replaces the apodization filter and DL0 does not remove any noise from k-space. This explains the lower SNR in the DL0 images compared to their non-DL counterparts as well as the lower CNR measured in the human subject images. For the prospective SOC imaging, DL25 also had a lower average CNR than the non-DL images although the median was higher, indicating that, in some cases, the DL25 is removing less noise than the apodization filter, thus leading to a lower CNR and bringing down the average. With DL100, the phantom SNR measurements varied among acquisition matrix sizes. DL100 ostensibly approaches removing all estimated noise which would yield unbounded SNR and highly unstable SNR measurements, which was consistent with our observations. The dependency of SNR increase with matrix acquisition size may be occurring due to the interaction between the specific noise realizations in each image and the noise estimation algorithm. The slight differences in expected noise characteristics among matrix sizes are also potential sources for variation in noise estimation.

Sharpness improvements were seen for all acquisition matrices with a substantial increase in sharpening observed when the matrix size increased from 320 × 320 to 356 × 356. Associated with this was an increase in reconstructed DL image size from 512 × 512 to 1024 × 1024. The DL reconstruction pipeline involves an interpolation of image data to remove the truncation artifact and increase the image sharpness [20]. The increase in image size was necessary to allow for sufficient interpolation of the existing data and was performed automatically in the prototype reconstruction as the matrix size exceeded 320 × 320. This additional interpolation resulted in the observed abrupt increase in sharpening. The amount of sharpening seen in the phantom was largely found to be independent of the noise level setting. This is consistent with the claimed decoupling of the noise estimation/reduction process from the sharpening process described by Lebel [20]. In addition to confirming the decoupling of sharpening and denoising this work demonstrates that it persists across multiple acquisition matrix sizes.

While DL improved artifact scores for SOC imaging, scores worsened when used with HR imaging. In both scenarios, the radiologists noted that artifacts were “intensified” in some cases after application of DL. The profile through the artifact suggested that a combination of higher artifact and lower background signal intensity led to the increased conspicuity of artifacts. Previously published work reported similar worsening of artifacts with the same DL reconstruction specifically citing pulsation and motion ghost artifacts [28]. Worse artifact performance is consistent with the expected performance of the DL algorithm since it was explicitly trained not to reduce image artifacts other than those due to data truncation [20]. HR imaging may have worsened already existing artifacts (such as ghosting) or introduced new artifacts which are then “intensified” by the DL. This idea is supported by our data in which HR imaging showed an initial decrease in artifact scores and a further decrease after DL was applied.

It is worth noting that the “intensified” artifacts were not necessarily detrimental to image interpretation. Although more prominent with DL than without, artifacts retain a familiar appearance. Furthermore, readers reported that the “intensified” artifacts were sometimes easier to identify as being artifactual and consequently ignored. However, this may not be the case for all specific clinical scenarios. Because it is impossible to fully eliminate artifacts from raw data, it is prudent to consider what types of artifacts are to be expected with a particular sequence and how they may be impacted by the DL reconstruction.

As a whole, radiologists in this study preferred the DL reconstruction in the context of SOC imaging. However, a differential image preference was observed between reader 1 and reader 2 when viewing HR images. Reader 1 generally preferred the HR DL images and reader 2 preferred non-DL images citing over-smoothing of features and a “fake look” to the DL images. However, reader 2 still gave DL images higher scores in the reader study. The reader’s proclivity for non-DL images appears to be a simple difference in preference and not indicative of any image quality issues. It should be noted that reader 1 participated in both reader studies, while reader 2 only participated in the HR study. Reader 1 was therefore more familiar with the appearance of the DL reconstructions at the time of review. According to the global–focal search model of image perception, radiologists have an expert’s schemata, that is, a mental picture of what the typical MR images are expected to look like [29]. The DL reconstructed images may be too different from reader 2′s expert schemata to be accepted as “real” images. Concerns about “over smoothing” could be addressed in part by reducing the noise reduction parameter of the DL reconstruction. In this work, the reader study used DL75 which indicates a high amount of denoising. This was carried out based on previous work showing promising results with DL75 [21,22,23] and to match the highest denoising level available with in the commercial version of the reconstruction. Wang et al. had radiologists compare denoising levels in prostate imaging and found a preference for DL75 [23]. Koch et al. found radiologist preference was split between DL50 and DL75 [24]. Decreasing the noise reduction parameter in our case may help to alleviate some of the concerns raised by reader 2. Since additional reconstructions with other noise parameters are “cheap”, they require no collection of additional acquisition data, it may be astute to provide all the denoising levels in a clinical setting, allowing for radiologists to browse the different reconstructions based on their preferences or based on the DL performance on a case-by-case basis.

For clinical interpretation, T2w breast images are viewed in context with the T1w DCE sequence. However, in this study, radiologists were asked to perform their assessment of the T2w images independently of T1w DCE images. This work focused on quantitative and qualitative measures of image quality and not on clinical performance. As such, an assessment of diagnostic performance was not obtained. Instead, this work provides the foundation of quality improvements to justify further study of DL’s impact on clinical interpretation and decision making. Further evaluation of the DL is needed to assess diagnostic performance. Another limitation is the receive coil array used for phantom studies was not identical to the breast coil used for human subjects imaging. Therefore, the exact SNR increase measured in phantom may not be directly replicated in in vivo imaging. However, the CNR increase in human subjects was around 2.0 times for DL75 which is on par with the SNR increase in phantom for DL75 and matrix size of 448 × 448 which was 2.8. The lower increase may be due to coil differences, noise characterization differences, or the difficulty in estimating noise in vivo by the reconstruction algorithm. Finally, this study was performed exclusively at 3 T and did not include 1.5 T MR imaging. DL may provide additional benefit to imaging at 1.5 T due to the overall increase in image noise expected at lower field strengths. Future work should include imaging at 1.5 T as well.

## 5. Conclusions

The vendor-supplied DL-based reconstruction performed well in the setting of T2w breast MRI and appears to be compatible with multiple breast imaging protocols. It allowed for superior image quality in SOC and HR protocols according to quantitative and qualitative metrics, while maintaining hyperintense signal in T2-bright features. Radiologists generally preferred DL SOC images due to an increase in sharpness and a decrease in noise. However, image preference in the HR setting was split due to some radiologists finding the images too smooth and appearing unreal. Further studies are necessary to assess the impact of a DL reconstruction on diagnostic accuracy. Overall, the DL shows promise for improving T2w image quality in clinical breast MRI.

## Figures and Tables

**Table 1:** T2w acquisition sequence.

Parameter	Scanner 1		Scanner 2	
T2w Acquisition	SOC	HR	SOC	HR
Plane	Axial	Axial	Axial	Axial
Type	2D FSE	2D FSE	2D FSE	2D FSE
TR/TE (ms)	3500/85	3500/85	4000/85	4000/85
Acquisition Matrix	288 × 288	448 × 448	228 × 228	448 × 448
FOV (cm)	32–36	32–36	32–36	32–36
Slice Thickness (mm)	2	2	2	2
PI Factor	3/SENSE	3/SENSE	3/GRAPPA	3/GRAPPA
ETL	16	16	16	16
Acquisition Time (s)	247	308	237	284

T2w—T2-weighted; SOC—standard of care; HR—high resolution; FSE—fast spin echo; FOV—field of view; PI—parallel imaging; ETL—echo train length.

**Table 2:** Qualitative Likert scoring criteria.

Score	Overall Image Quality
5	Excellent: no artifacts and anatomical detail well visualized
4	Good: minor artifacts, some blurriness, no impact on diagnostic capability
3	Fair: major or multiple minor artifacts, blurriness, no impact on diagnostic capability
2	Poor: multiple major or minor artifacts, loss of detail, impact on diagnostic capability
1	Non-diagnostic: severe artifacts, and complete loss of anatomical detail

## Data Availability

Data used in this work are available on request at URL: https://radiology.wisc.edu/research/data/ (accessed on 3 August 2023). The signing of a data use agreement will be needed.

## Supplementary Materials

The following supporting information can be downloaded at: https://www.mdpi.com/article/10.3390/tomography9050152/s1, Table S1: Reader Study Rubric; Figure S1: Phantom Edge Sharpening; Table S2: T2-weighted Image Preference by Reader.
