# Supplementary material for: Evaluation of a Deep Learning Reconstruction for High-Quality T2-Weighted Breast Magnetic Resonance Imaging
Source: Tomography. 2023 Oct 18;9(5):1949–64. doi: 10.3390/tomography9050152 (PMC10611328; doi:10.3390/tomography9050152)
Supplement: Supplementary file 1 [file tomography-09-00152-s001.zip › tomography-2570126-supplementary.pdf]

Reader Study Rubric

Minor artifact-> the artifact does not affect areas of interest, the artifact is easily read through, the artifact has only limited effect.

Major artifact-> the artifact obscures the area of interest; the artifact greatly impacts the visualization of tissues.

Impact on Diagnostic Capability-> The reader feels that the content of a section (SNR, artifact, etc.) is impacting their ability to confidently view or identify areas of interest in the image.

Table S1: Rubric used by radiologist readers to score image quality.

Score	SNR	Artifacts	Perceived Sharpness	Overall
5	Excellent	Excellent: no artifacts	Excellent: edges are sharp and distinct	Excellent: no artifacts and anatomical detail well visualized
4	Good	Good: minor artifacts, no impact on diagnostic capability	Good: edges seen clearly, slight blurriness, no impact on diagnostic capability	Good: minor artifacts, some blurriness, no impact on diagnostic capability
3	Fair	Fair: major or multiple minor artifacts, no impact on diagnostic capability	Fair: some blurring, loss of anatomical detail, no impact on diagnostic capability	Fair: major or multiple minor artifacts, blurriness, no impact on diagnostic capability
2	Poor	Poor: multiple major or minor artifacts, impact on diagnostic capability	Poor: blurring, loss of anatomical detail, impact on diagnostic capability	Poor: multiple major or minor artifacts, loss of detail, impact on diagnostic capability
1	Non-diagnostic	Non-diagnostic: image is unreadable	Non-diagnostic: features are blurred beyond recognition	Non-diagnostic: severe artifacts, and complete loss of anatomical detail

Table S2: T2-weighted Image Preference by Reader.

SOC Reader Preference			
	SOC, non-DL	SOC, DL	No Preference
Reader 1	1	18	-
Reader 2	-	19	-
Reader 3	3	15	1
Reader 4	-	19	-

HR Reader Preference			
	SOC, non-DL	HR, non-DL	HR, DL
Reader 1	2	2	50
Reader 2	23	31	-

SOC: Standard of Care, HR: High Resolution, DL: Deep Learning.

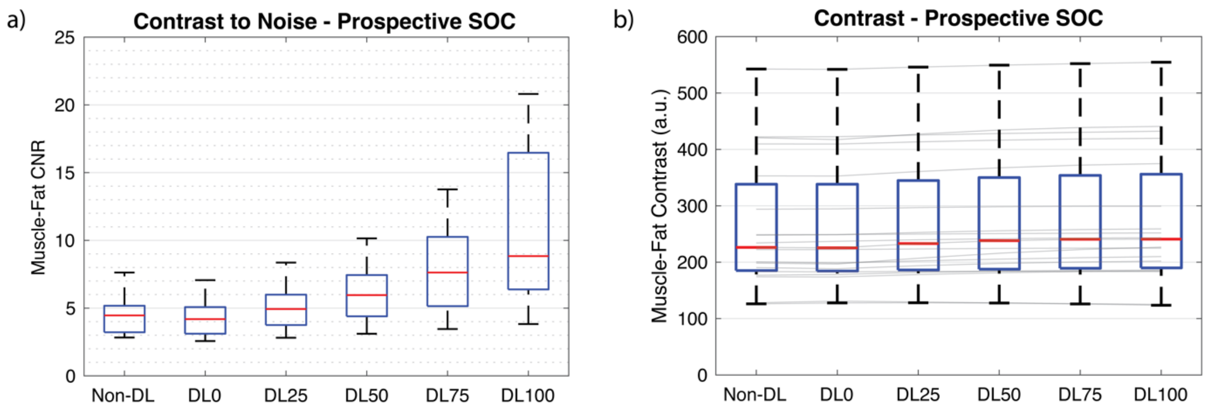

Figure S1. a) Muscle-fat contrast-to-noise ratio (CNR) increased when the deep learning reconstruction (DL) was applied with a noise reduction greater than 25% (DL25). Higher levels of denoising produced higher CNR. Use of DL without denoising (DL0) slightly reduced the CNR. Box plots show the distributions of CNR measurements for 19 retrospectively collected standard-of-care (SOC) T2w breast exams. The red horizontal line represents the median value. An outlier point existed for DL100 at a CNR of 46, however it was cropped to preserve the readability of the plot. **b)** Muscle-fat contrast (rather than contrast to noise) shows the contrast remains consistent across all images regardless of DL. The thin faint horizontal lines represent the 19 individual exams and show that contrast for each exam was constant across all reconstructions.
